# Psychiatric Disorders and Personality Profiles of Middle-Aged Suicide Attempters with No Evidence of Specific Psychopathological Profiles Referred to an Emergency Department

**Published:** 2017-10

**Authors:** Serge Brand, Mehri Nejat, Mohammad Haghighi, Alireza Rahimi, Leila Jahangard, Dena Sadeghi Bahmani, Hafez Bajoghli, Edith Holsboer-Trachsler, Mohammad Ahmadpanah

**Affiliations:** 1 University of Basel, Psychiatric Hospital of the University of Basel, Center for Affective, Stress and Sleep Disorders, University of Basel, Basel, Switzerland.; 2 University of Basel, Department of Sport, Exercise, and Health, Division of Sport and Psychosocial Health, Basel, Switzerland.; 3 Kermanshah University of Medical Sciences (KUMS), Department of Psychiatry, Substance Abuse Prevention Center and Sleep Disorder Prevention Center, Kermanshah, Iran.; 4 Behavioral Disorders and Substances Abuse Research Center, Hamadan University of Medial Sciences, Hamadan, Iran.; 5 Iranian National Center for Addiction Studies (INCAS), Tehran University of Medical Sciences, Tehran, Iran.

**Keywords:** *Suicide Attempts*, *Middle-Aged Patients*, *Psychopathology*, *Sociodemographic Dimensions*, *Emergency Unit*

## Abstract

**Objective:** The present study aimed at assessing the sociodemographic and psychiatric characteristics of 40 to 65 year- old suicide attempters, who were referred to an emergency department within 4 hours of the attempt.

**Method:** A total of 93 suicide attempters (Mean age=46.59 years) who were referred to an emergency department, were assessed in this study. Patients completed questionnaires covering sociodemographic data, personality traits, mood, and impulsivity. Psychiatric status of the patients was rated by experts.

**Results:** Experts rated 85 (92.4%) of the suicide attempters as having a psychiatric disorder. Based on self-ratings and compared to normative data, 42 (46.6%) patients were psychopathologically ill. It was found that suicide attempts were not related to impulsive personality traits, mood disorders, sociodemographic patterns, or gender (gender-ratio: 1:1.58; f: m).

**Conclusion:** The pattern of results suggests that further unknown factors were involved in pushing people to attempt suicide.

As regards to the rate of suicide in Iran, recent estimations reported a prevalence of 5.3/100 000, with a higher rate in males (7 100 000) than in females (3.6 100 000) (1). Suicide and suicidal behavior demand particular attention, especially in the social environment of the attempters, as it causes dramatic suffering for those who commit or attempt suicide. Socioeconomic and psychiatric explanations have been advanced for the complex phenomenon of suicide and suicidal behavior. 

Socioeconomic approaches include the proposition that at a macro level, suicide and suicidal behavior may increase during economic crises. Recent data from Greece (2) have not supported this notion, when factors such as psychiatric disorders and social environment are taken into account. However, on a micro level, economic constraints have been reported as one of the factors in attempted suicide in Iran (3).

The developing countries in West Asia including Iran are in the process of nutrition transition and prevalence of obesity-related diseases including cardiovascular disease, Type 2 diabetes, hypertension, and cancers (18-19). In Iran, nutrition transition is taking place with accelerated demographic change, urbanization, and social expansion in spite of insignificant economic Next, Next, Evidence from psychiatry points to a close association of suicide and suicidal behavior with psychiatric disorders (4). Ajdacic-Gross (5) reported the following increases in suicide risk (compared to healthy controls): 200-fold increase in patients immediately after discharge; 50-fold increase in patients while in psychiatric hospitals; 90-fold increase in those patients with more than one psychiatric diagnose within the last four week; and 10-25-fold increase in patients with different psychiatric diagnoses. With respect to completed suicides, in their meta-analysis, Arsenault-Lapierre et al. (4) reported the following: (a) a mental disorder was diagnosed in 87% of the 3275 patients included in the studies; (b) with respect to gender, diagnoses of substance-related problems, personality disorders, and childhood disorders were more common among male suicides, while affective disorders including depressive disorders were more common among female suicides.

Additionally, there is some evidence that irrespective of psychiatric diagnoses, impulsive behavior is related to an increased risk of suicide (suicide attempt). Menon et al. (6) and Pompili et al. (7) reported impulsivity, along with the intake of antidepressants, to be the best predictor of suicidal intent. Taking these observations into account, in the present study we aimed at finding to what extent characteristics of impulsivity (patients with bipolar I disorders, or patients with narcissistic, antisocial, sadistic, or compulsive personality traits) might be observable among a sample of suicide attempters who were referred to an emergency department. Overall, while there is evidence that psychiatric illnesses and risk of suicide and suicide behavior are related, evidence is also accumulating that the fit is imperfect. Milner et al. (8) conducted a systematic review and meta-analysis of the relationship between completed suicide and psychiatric disorders, and they found that some publications had identified an absence of any psychiatric diagnosis in up to 67% of the cases (according to the DSM-IV or ICD-10). They further found that 37.1% of the suicide cases had no psychiatric condition (i.e., personality disorder or accentuated personality) across the reviewed studies. 

Gender differences were found as a sociodemographic characteristic of suicide and suicide attempters. Males are involved in more completed suicides than females, but it was found that the latter make more attempts, with a ratio of 3:1 (1, 4, 5, 9-11). 

Last, the present study expands upon previous studies in Iran (1, 3, 12, 13) in two aspects. First, previous results on suicide behavior in Iran have relied on experts’ ratings alone (1, 3, 12, 13). In the present study, we assessed patients’ view of their psychological and psychopathological state. To this end, patients completed a self-rating questionnaire at a maximum of 4 hours after referral to the emergency department, following a suicide attempt. This self-rating questionnaire focuses on a variety of psychopathological personality dimensions, allowing a deepened insight into patients’ points of view as to why a suicide was attempted. Second, we specifically investigated patients who were middle-aged (40-65 years), as this age range of suicide attempters has been under investigation thus far, while on the other hand, it covers 19.47% of the Iranian population.

The following three hypotheses were formulated: First, following Arsenault-Lapierre et al. (4) and Milner et al. (8), we expected that 67% to 87% of patients would meet the DSM-IV criteria for psychiatric disorders, as diagnosed by psychiatrists and clinical psychologists. Second, we expected higher rates of such disorders, compared to the prevalence of other psychiatric disorders. Third, following Milner et al. (8), we expected higher rates of psychopathological personality traits in the self-ratings, when compared to cut-off-values from normative data.

## Materials and Methods

*Procedure*

Patients who were referred to the emergency department of a general hospital in Hamadan (Iran) following a suicide attempt within the previous 4 hours were approached. All patients were informed about the aim of the study, and the voluntary basis of participation. Furthermore, all participants were assured about the anonymity of the data, and written informed consent was requested. Then, a psychiatrist or clinical psychologist conducted a structured psychiatric interview (see below), and patients completed a self-rating questionnaire on psychopathology (see below). The ethical committee of Hamadan University of Medical Sciences (Hamadan, Iran) approved the study; furthermore, this study was in accordance with the Declaration of Helsinki.

*Sample*

A total of 93 patients (mean age: M = 46.59 years; SD = 6.73; 36 (38.7%) females) took part in the study. Inclusion criteria were as follows: 1. suicide attempt within the last 4 hours; 2. age 40 to 65 years; 3. signing written informed consent; 4. willing and able to submit to a clinical interview; and 5. completing a self-rating questionnaire on psychopathology (see below). Exclusion criteria were as follow: 1. status of continuous suicidality; 2. status of psychosis. 

A total of 156 patients were approached, of whom 45 (28.8%) did not meet the inclusion and exclusion criteria, 8 (5.1%) refused participation, and 10 (6.41%) withdrew after the beginning of the study. Thus, 59.6 % (N= 93) completed the study. Gender ratio of non-participants was approximately 1:1.

*Clinical Assessment*

First, sociodemographic data were assessed (age, gender, civil status, highest level of education achieved, and employment status), and the method employed in the suicide attempt was determined (poisoning, hanging/suffocation, drug overdose, and other techniques such as cutting, or electrical shock). Next, to diagnose patients’ psychiatric disorders according to the DMS-IV-TR (14), a clinical interview was conducted based on the SCID-I (Structured Clinical Interview for DSM-IV Axis I Disorders (15); Persian translation and validation: Sharifi et al., 2009 (16). Finally, patients completed a questionnaire covering dimensions of psychopathology (self-rating; Millon Clinical Multiaxial Inventory –III; (17).

As mentioned, to assess possible psychopathological traits from the patients’ view, patients were asked to complete the 175-item Millon Multiaxial Inventory-III (17) (Persian validated version (18)), a self-rating instrument to identify personality disorders and psychiatric problems. The following personality dimensions were assessed: schizoid, avoidant, depressive, dependent, histrionic, narcissistic, antisocial (aggressive), sadistic, compulsive, negativistic (passive-aggressive), masochistic (self-defeating), schizotypal, borderline, and paranoid. The following clinical conditions were assessed in accordance with Axis I diagnoses of the DSM-IV: anxiety, somatoform syndromes, bipolar: manic, dysthymia, alcohol dependence, drug dependence, PTSD, thought disorders, major depression, and delusional disorder. Answers were given as true-false, with higher scores reflecting a more extreme position on a dimension. Furthermore, the following cut-off values were indicated: <59 percentiles: below psychiatric threshold; 60 to 74 percentiles: psychiatric population; 75 to 79 percentiles: presence of a particular psychiatric trait or syndrome; >80 percentiles: presence of a disorder or prominence of a syndrome 

*Statistical Analysis*

First, a series of X^2^-tests was performed to calculate possible associations between gender and sociodemographic variables. Next, a series of t-tests was performed to calculate gender differences in psychopathology (as self-rated). With performing further X^2^-tests, we calculated the associations between gender and psychiatric diagnoses (as rated by experts). 

The level of significance was set at alpha ≤.05 and statistical analysis was performed with SPSS® 22.0 (IBM Corporation, Armonk NY, USA) for Apple Mac®.

## Results

*Sociodemographic Variables*

Table 1 reports all statistical indices for the sociodemographic variable. 

With respect to gender differences, compared to males, more female patients reported to be married, rather than single, and they also reported not being in paid employment, and if employed, they did not hold a management position.

*Psychopathology (self-rating and experts’ ratings)*

Table 2 demonstrates all the statistical indices (descriptive and inferential statistics) related to dimensions of psychopathology

*Incidence of psychiatric disorder as rated by experts*

Table 2 shows that 8 patients (8.6%) had no diagnosis. Conversely, experts made psychiatric diagnoses in 85 cases (91.4%).

*Incidence of psychiatric characteristics as self-rated by patients*

Table 2 demonstrates that 50 patients (n = 50.6; 54.4%) did not reach psychopathologically high percentiles (percentiles of 59 or higher), while 10 patients (n = 9.5; 7.1%) reported psychopathological characteristics as a primary problem (percentiles of 80 or higher).

Table 2 shows that personality traits characterized by impulsivity (narcissistic, antisocial, aggressive, compulsive) were not predominant; 21 patients (21.6%) reported such psychopathological characteristics as a primary problem, while for the other 73 patients (88.4%) personality traits characterized by impulsivity were not predominant. 

*Gender Ratio*

We observed a female/male ratio of 1:1.58. 

## Discussion

The key findings of the present study were that among middle- aged suicide attempters, who were referred to an emergency room, 46.6% (self-rating) to 91.4% (experts’ ratings) were psychopathologically ill, while no other patterns, whether sociodemographic or illness-related, were observed. The present findings add to the current literature in suggesting that beyond psychopathology, further unknown factors seem to be involved in suicidal behavior. Three hypotheses were formulated.

Our first hypothesis was that psychiatric diagnoses would be in the range 67% to 87%. In the present sample, the rate of psychiatric diagnosis was 91.4%. Whereas this incidence is descriptively higher, the general direction is that suicide attempts are related to psychiatric disorders. The present pattern of results is, therefore, in accordance with numerous previous studies (4, 5, 9, 10). On the other hand, we found that from the patients’ view, a much lower level of psychiatrically predominant characteristics was reported as a primary problem. 

With the second hypothesis, we expected higher rates of personality characteristics related to impulsivity (narcissistic, antisocial, aggressive, and compulsive personality traits) and mood disorders, but this was not confirmed. However, no specific psychiatrically predominant characteristic could be observed (Table 2). Therefore, we speculate that suicide attempts were related to further factors beyond impulsivity. Accordingly, the present data did not fit the previous results as reported by Menon et al. (6) and Pompili et al. (7).

Our third hypothesis was that self-ratings for psychopathological features would be in percentiles above the cut-offs for psychopathology (see Table 2); however, this hypothesis was not confirmed, as 54.4% of the patients’ ratings were below psychiatric significance. 

**Table 1:** Descriptive overview of socio-demographic variables, for the entire sample, and separately for gender.

	**Entire sample**	**Sample**		**Statistics**
		**Female patients**	**Male patients**	
N	93	36	57	
Age (years) M (SD)	46.59 (6.73)	47.92 (7.52)	45.75 (6.10)	t(91) = 1.52
	N (%)	N (%)	N (%)	
**Civil status**				
Married	75 (80.6)	34 (94.4)	41 (71.9)	X^2^(N = 93, df = 3) = 10.44***
Divorced	0 (0)	0 (0)	0 (0)	
Single	19 (15.1)	0 (0)	14 (24.6)	
Widowed	4 (4.3)	4 (5.6)	0 (0)	
				
**Highest education**				
Primary school	32 (34.4)	15 (41.7)	17 (29.8)	X^2^(N = 93, df = 2) = 4.02
Secondary school	33 (35.5)	11 (30.6)	22 (38.6)	
Diploma	21 (22.6)	9 (25)	12 (21)	
University	3 (3.2)	1 (2.8)	2 (3.5)	
No answer	4 (4.3)	0 (0)	4 (7)	
				
**Job categories**				
Unemployed	10 (10.8)	0 (0)	10 (17.5)	X^2^(N = 93, df = 2) = 71.63***
Housewife	30 (32.3)	30 (83.3)	0 (0)	
Retired	9 (9.7)	2 (5.6)	7 (12.3)	
Worker	33 (35.5)	2 (5.6)	31 (54.4)	
Management	11 (11.8)	2 (5.6)	9 (15.8)	
				
**Type of suicide ** **attempts**				
Poisoning	15 (16.1)	8 (8.6)	7 (7.5)	
Hanging/suffocation	12 (12.9)	0 (0)	12 (12.9)	
Drug overdose	54 (58.1)	28 (30.1)	26 (28.0)	
Various techniques (cutting, traffic accidents, electrical shock)	12(12.9)	0 (0)	12 (12.9)	X^2^(N = 93, df = 3) = 20.44***

*** Notes: = p < .001.

**Table 2:** Descriptive and inferential statistics of MCMI-III Scale Scores and psychiatric diagnoses for the entire sample and separately for female and male patients, and compared to normative data.

				**Samples**				**Statistics**												
	**Entire ** **sample**		**Female ** **patient** **s**			**Male ** **patients**					**Number ** **and % of ** **patients ** **below ** **median of ** **psychiatri** **c patients** **< BR 59**		**Number ** **and % of ** **patients ** **above ** **median of ** **psychiatric ** **patients** **BR 60 - 74**		**Number and ** **% of patients ** **with presence ** **of a particular ** **characteristic** **BR 75 -79**			**Number and % ** **of patients with ** **predominant ** **characteristic ** **as primary ** **problem** **> BR 80**		
N		93			36		57		t-tests											
		M SD			M SD		M SD													
							
Schizoid	53.62 (18.63)			55.19 (17.39)			52.63 (19.54)		0.60		44 (47.31)		40 (43.0%)		2 (2.6%)		2 (2.6%)			
Avoidant	52.70 (15.59)			50.81 (11.42)			53.89 (17.71)		0.93		56 (59.3)		33 (35.5%)		2 (2.6%)		2 (2.6%)			
Depressive	65.13 (24.75)			70.86 (19.63)			61.51 (27.04)		1.80 (*)		29 (31.2)		33 (35.5%)		4 (4.3%)		27(29.0%)			
Dependent	46.12 (22.75)			46.44 (19.97)			54.91 (24.51)		0.11		62 (66.6)		21 (22.6%)		1 (1.1%)		9 (9.7%)			
Histrionic	54.84 (23.42)			55.28 (23.99)			54.56 (23.26)		0.14		46 (48.3)		28 (30.1%)		6 (6.5%)		14(15.1%)			
Narcissistic	38.96 (26.74)			33.13 (19.45)			42.53 (30.07)		1.64		74 (79.5)		9 (9.7%)		1 (1.1%)		9 (9.7%)			
Antisocial	48.87 (19.93)			43.86 (19.19)			52.04 (19.90)		1.96 *		64 (68.8)		23 (24.7%)		1 (1.1%)		5 (5.4%)			
Aggressive	47.24 (18.60)			58.22 (18.09)			46.61 (19.05)		0.40		66 (70.9)		22 (23.7%)		2 (2.6%)		3 (3.2%)			
Compulsive	42.81 (23.54)			44.64 (21.10)			41.65 (25.08)			0.59	65 (69.9)		22 (23.7%)		2 (2.6%)	4 (4.3%)				
Passive-aggressive	55.55 (16.34)			58.75 (13.88)			53.53 (17.53)			1.51	41 (44.1)		47 (50.5%)		4 (4.3%)	1 (1.1%)				
Self-defeating	49.76 (19.96)			53.58 (16.25)			47.35 (21.62)			1.48	54 (58.1)		38 (40.9%)		0 (0)	1 (1.1%)				
Schizotypal	47.42 (17.94)			46.36 (16.14)			48.09 (19.09)			0.45	68 (73.1)		24 (25.8%)		1 (1.1%)	0 (0)				
Borderline	53.10 (19.12)			45.78 (20.27)			52.04 (18.48)			0.67	54 (58.1)		32 (34.4%)		4 (4.3%)	3 (3.2%)				
Paranoid	51.08 (19.02)			49.75 (18.31)			51.91 (19.57)			0.53	44 (52.2)		41 (44.1%)		2 (2.6%)	1 (1.1%)				
Anxiety	62.28 (17.86)			60.39 (13.24)			60.32 (20.11)			1.34	25 (26.8)		50 (53.8%)		9 (9.7%)	9 (9.7%)				
Somatoform	54.72 (19.55)			56.25 (19.50)			53.75 (19.69)			0.60	40 (43.0)		47 (50.5%)		4 (4.3%)	2 (2.6%)				
Bipolar	42.81 (22.24)			41.06 (21.71)			43.91 (22.69)			0.60	69 (70.7)		20 (21.5%)		2 (2.6%)	2 (2.6%)				
Dysthymia	56.74 (19.97)			60.92 (14.57)			54.11 (22.45)			1.62	36 (38.7)		41 (44.1%)		8 (8.6%)	8 (8.6%)				
Alcohol	40.67 (21.09)			36.28 (19.51)			43.44 (21.74)			1.61	73 (78.5)		17 (18.3%)		0 (0)	3 (3.2%)				
Drug	42.62 (24.94)			32.64 (22.94)			48.95 (24.25)			2.23 **	64 (68.8)		21 (22.6%)		3 (3.2%)	5 (5.4%)				
PTSD	50.34 (24.94)			53.94 (22.05)			48.07 (24.89)			1.16	46 (49.5)		34 (36.6%)		8 (8.6%)	5 (5.4%)				
Thought Disorder	66.75 (18.70)			67.36 (17.46)			66.37 (19.59)			0.25	22 (23.7)		33 (35.5%)		17 (18.3%)	21(22.6%)				
Major Depression	67.89 (22.21)			71.08 (20.26)			65.88 (23.30)			1.10	24 (25.8)		26 (28%)		10 (10.8%)	33(35.5%)				
Delusional Disorder	47.29 (21.67)			44.37 (21.77)			49.09 (21.60)			1.01	56 (60.2)		31 (33.3%)		4 (4.3%)	2 (2.6%)				
Mean SD	N (%)										50.6 (54.4)		40.72 (43.8)		7.89 (5.8)	9.5 (7.07)				
				N (%)			N (%)													
Psychiatric diagnoses (DSM-IV); experts’ ratings																				
No diagnose	8 (8.6)			4 (4.3)			4 (4.3)													
Schizophrenia	4 (4.3)			2 (2.2)			2 (2.2)							Gender x Diagnosis X^2^(N=93, df=5)=22.39***						
Mood disorders	55 (59.2)			20 (21.5)			35 (37.6)													
Anxiety disorders	10 (10.75)			6 (6.5)			4 (4.3)													
Substance- induced disorders	10 (10.75)			0 (0)			10 (10.8)													
Adjustment disorders	6 (6.45) 93 (100)			6 (6.5) 36 (38.7)			0 (0) 57 (61.3)													

Notes. PTSD = Posttraumatic Stress Disorder;

* = p < .05;

** = p < .01;

*** = p < .001.

Again, the data were at odds with previous results such as those of Milner et al (8). Moreover, the quality of the data did not allow a deeper insight into the underlying cognitive-emotional processes; thus, the patients might have felt ashamed of revealing their weaknesses, or they might have felt unable to properly and thoroughly complete a questionnaire shortly after having tried to end their lives.

## Limitations

The novel findings of this study should be balanced against the following limitations. First, the quality of the data did not allow us to identify specific life circumstances or triggers. Specifically, impulsive behavior and critical life events increase the risk of suicide attempts. Critical life events are, for instance, continually increasing or repetitive psychosocial difficulties such as loss of a loved person, or chronic somatic illnesses. Second, we asked about the civil status (single, married, divorced, or widowed) as a proxy for social interaction and commitment, although civil status does not reflect the quantity and quality of a person’s social relationships. In this respect, low levels of interpersonal trust was the third leading risk factor for suicide attempts, after major depressive disorders and previous suicide attempts (2). This observation was particularly pertinent given Nazarzadeh et al.’s (3) report that family conflicts and marital conflicts were among the most frequent causes of attempted suicide, followed by economic constraints and educational failure. Third, to the best of our knowledge, in the context of research on suicidal behavior, this study was unique in asking patients not later than 4 hours after admission to an emergency room to participate in a psychiatric interview and to complete a questionnaire. However, such an approach also carries the risk that data are gathered under unfavorable psychological conditions. Specifically, it is highly likely that the cognitive performance would be compromised given such mental states as anxiety (19) and stress (20). Therefore, the results from this self-rating questionnaire should be interpreted with caution. 

In addition, not all suicide attempters were referred to an emergency department, and not all were referred to the emergency department of the general hospital at which this study took place. Thus, the sample of suicide attempters may be biased and not representative of suicide attempters as a whole.

## Conclusion

Suicide attempters aged 40 to 65 years who were referred to an emergency department were psychopathologically ill, both from patients and from experts’ view. Suicide and attempted suicide should be a topic of routine counseling of patients with psychiatric disorders to reduce the risk of suicide.
